# Initiating buprenorphine to treat opioid use disorder without prerequisite withdrawal: a systematic review

**DOI:** 10.1186/s13722-021-00244-8

**Published:** 2021-06-08

**Authors:** K. K. Adams, M. Machnicz, D. M. Sobieraj

**Affiliations:** 1 University of Connecticut School of Pharmacy, 69 N Eagleville Rd Unit 3092, Storrs, CT 06269 USA; 2 Hartford Hospital, Hartford, USA

**Keywords:** Buprenorphine, Initiation, Opioid use disorder, Withdrawal, Microdosing

## Abstract

**Background:**

Opioid withdrawal symptoms prior to buprenorphine initiation may be intolerable and as a result, alternative strategies have emerged. We aim to systematically review the efficacy and safety of buprenorphine initiation that aims to omit prerequisite withdrawal.

**Methods:**

We conducted a systematic literature search of MEDLINE and CENTRAL from 1996 through April 10, 2020, augmented with searches in Google Scholar and www.clinicaltrials.gov. A study was included if it was in patients with substance use disorder or chronic pain that were taking a full mu opioid agonist and transitioning to buprenorphine without preceding withdrawal, and reported withdrawal during initiation as an outcome. Two investigators independently screened citations and articles for inclusion, collected data using a standardized data collection tool, and assessed study risk of bias.

**Results:**

We included 15 case reports/series, reporting 24 unique cases, in our qualitative synthesis. No controlled studies were identified. Microdosing and bridging with a buprenorphine patch were the most common strategies reported. Transition to buprenorphine with complete cessation of opioid agonists was achieved in 87.5% (n = 21) of cases. Withdrawal during initiation occurred in 58.3% (n = 14) of cases, two of which were at least moderate in severity.

**Conclusion:**

Buprenorphine initiation strategies that omit prerequisite withdrawal have emerged. Low quality evidence from case reports suggests withdrawal during initiation is common but most often mild in severity. There is an unmet need for controlled studies to inform their efficacy and safety compared with traditional strategies, including outcomes during initiation and in the long-term.

**Supplementary Information:**

The online version contains supplementary material available at 10.1186/s13722-021-00244-8.

## Introduction

Buprenorphine is a first-line treatment for opioid use disorder (OUD) that decreases all-cause mortality [1] and expansion of buprenorphine treatment is associated with a decline in heroin overdose deaths [2]. Buprenorphine has a favorable safety profile due to its ceiling effect for respiratory depression [3] and is an effective treatment for chronic pain in patients unresponsive to other opioids [4]. Of note, sublingual buprenorphine for chronic pain is off-label according to the Food and Drug Administration (FDA) and may provoke inquiry during a Drug Enforcement Administration (DEA) audit of buprenorphine prescriptions [5]. Despite its benefits, a major challenge to using buprenorphine lies in its partial agonist properties that can lead to precipitated withdrawal if administered while full opioid agonist is still bound to the mu receptor [6]. Precipitated withdrawal is physically uncomfortable and may lead to increased rates of treatment dropout or relapse [7, 8]. Complications from precipitated withdrawal, such as involuntary limb movement, can be distressing to patients and difficult to treat [9, 10] and may deter future initiation attempts [11]. While rare, precipitated withdrawal can lead to emergency room admissions [12] and life-threatening complications [13]. Clinicians whose patients experience precipitated withdrawal may be discouraged to offer this life-saving medication to patients in the future [11]. Some speculate that replacement of heroin with fentanyl in illicit drug markets may create additional barriers to buprenorphine initiation [14]. Additionally, anecdotal reports note increased incidence of precipitated withdrawal during buprenorphine initiation, likely due to fentanyl’s lipophilicity and increased volume of distribution [14].

To avoid precipitated withdrawal, buprenorphine is usually initiated after an opioid free interval when mild to moderate withdrawal symptoms are present [15]. Not all patients can tolerate the physical distress, and this can become an additional barrier to buprenorphine treatment or a cause of opioid relapse [11, 16]. Alternative initiation strategies have emerged in practice with the goal of eliminating prerequisite withdrawal prior to buprenorphine initiation. We aim to systematically review the literature to identify these alternative buprenorphine initiation strategies and the efficacy and safety of these strategies compared to a traditional buprenorphine initiation.

## Methods

### Data sources and search

We completed this systematic review consistent with the Preferred Reporting Items for Systematic Reviews and Meta-analyses guidance [17]. We conducted a systematic literature search of MEDLINE and Cochrane Central Register of Controlled Trials from 1996 through April 10, 2020 (see Additional File 3). To augment our bibliographic database search, we searched Google Scholar on April 13, 2020 and reviewed potentially relevant citations from the prior year as well as forward citation tracking of relevant citations. We also reviewed the references of included studies. Finally, we searched www.clinicaltrials.gov for completed studies with results or ongoing studies to evaluate results that may be informative in the future.

### Study selection

Two investigators independently screened the title and abstract of each citation identified by the search and subsequently reviewed the full-text manuscript for final inclusion into the review. Discrepancies were resolved by discussion or a third investigator. A study (any design) was included if it (1) evaluated an alternative buprenorphine initiation strategy that aimed to avoid prerequisite opioid withdrawal, (2) was in patients with substance use disorder or chronic pain that were taking a full opioid agonist and (3) reported presence or absence of withdrawal during the initiation phase. Traditional initiation of buprenorphine includes an opioid-free period to establish mild to moderate prerequisite withdrawal, considered to be a minimum score of 5 or more on the Clinical Opiate Withdrawal Scale (COWS) [18–20]. Such studies were excluded. We defined alternative strategies aimed to avoid prerequisite withdrawal as those that either (1) overlapped the full opioid agonist and buprenorphine (omitting the opioid free period and thus omitting prerequisite withdrawal) or (2) reported a baseline COWS score of less than 5 (documenting lack of withdrawal symptoms). In the absence of a reported baseline COWS score, we estimated the maximal possible score using symptoms reported prior to the first buprenorphine dose and included reports with a score less than 5. In addition to withdrawal, additional outcomes of interest included severity of withdrawal, number of patients that fully transitioned from full opioid agonist to buprenorphine monotherapy, duration of the initiation period, number of patients with a deviation from the initiation protocol, and patient satisfaction. Post-initiation outcomes included the duration buprenorphine was continued and the number of patients that experienced the following: illicit drug use, overdose, and cravings.

### Data extraction and risk of bias assessment

Using a standardized data collection tool, two investigators independently collected the following data from included studies: patient characteristics including age, gender, substance use history, indication for buprenorphine; initiation regimen characteristics including the method used, setting, medication dosing details, duration of initiation period, use of ancillary medications; and information to assess risk of bias and our outcomes of interest.

We assessed the internal validity of included studies using a tool designed for case reports [21]. The tool includes eight questions across four domains: selection, ascertainment, causality and reporting. We answered each question as “yes” or “no” and summarize assessments. We omitted two of the original eight questions regarding challenge/re-challenge phenomenon and dose–response relationships intended for reporting of adverse events because this was not applicable to our topic. Internal validity was assessed for each study by two separate investigators with conflicts resolved through discussion.

## Results

Upon initial database and manual searches, we identified 1825 citations after duplicates were removed (see Additional file 1: Fig. S1). After citation screening, we reviewed 253 articles at the full text level. Fifteen articles met our inclusion criteria and were included in the systematic review, all of which were case series or case reports.

We evaluated risk of bias for each article (see Additional file 2: Appendix Table S1). The domains of selection and causality has most weaknesses. None of the articles described selection methods and if other patients had attempted the described protocol. Despite length of follow up being adequate in all reports, we judged “no” for ruling out potential alternative causes of withdrawal in all reports because they either provided supportive medications that could have influenced withdrawal symptoms, or did not comment whether supportive medications were given or not. Seven articles (47%) used adequate methods to ascertained exposures and outcomes, which we considered to be medical records and validated tools to measure withdrawal severity, respectively, as opposed to patient reported information. All but one article reported cases with sufficient detail to replicate them.

Twenty-four unique patient cases met our inclusion criteria (Additional file 2: Table S1). Two cases within the 15 articles were excluded because they did not report the presence or absence of withdrawal symptoms during buprenorphine initiation, our primary outcome [4, 11]. Most reports came from the United States or Canada and represented both genders of adults ranging from 19 to 72 years. In most cases, buprenorphine was initiated for OUD alone or in combination with pain in both inpatient and outpatient settings. There was a range of initiation strategies represented amongst the 24 cases including micro-dosing, bridging with a transdermal opioid, or a combination of multiple strategies. The most utilized strategy was the Bernese method of micro-dosing [16], followed by bridging with a buprenorphine patch. Rapid micro-dosing, bridging with a fentanyl patch, and combination strategies compromised a small fraction of cases.

Our primary outcome of interest was the number of cases that experienced any level of withdrawal during the initiation period; 14 of the 24 cases (58.3%) reported withdrawal symptoms and 2 of the 24 cases (8.3%) experienced withdrawal severity that was at least moderate (Additional file 2: Table S2). Three cases (12.5%) did not successfully transition from full opioid to buprenorphine monotherapy and continued full opioid agonists. Further synthesis of data is provide based on the specific initiation strategy.

### Micro-dosing

Microdosing was the most common initiation strategy reported (n = 13) (see Additional file 2: Appendix Table S2), more specifically the Bernese method (n = 10). All cases overlapped full opioid agonist with buprenorphine (median 7 days range 2 to 120 days) and four cases also reported baseline COWS scores less than 5 (range 0 to 4). Seven of the 13 cases utilized an initial buprenorphine dose of 0.5 mg while the other 6 cases utilized smaller initial buprenorphine doses, as low as 0.2 mg. While exact doses and rates of titration varied between cases, the majority followed a general framework of increasing the total daily buprenorphine dose by 50–100% per day. Fewer than half of the cases achieved a buprenorphine dose of 16 mg or greater at the end of initiation, a dose that has been associated with long term retention [22]. Most cases (85%) completed initiation within 2 weeks (median 8 days, range 3 to 120 days). Seven of 13 cases (54%) reported withdrawal symptoms of any severity during initiation. One case experienced withdrawal of moderate severity. The frequency of withdrawal was similar, regardless if the initial buprenorphine dose was ≥ 0.5 mg or < 0.5 mg (approximately 50% in each group).

### Buprenorphine patch bridging

The second most common initiation strategy was bridging with the buprenorphine patch (10 or 20 ug/hr) to sublingual buprenorphine (n = 7) (see Additional file 2: Appendix Table S3). The first sublingual buprenorphine dose ranged from 2 to 8 mg and was given between days 2 and 4 (median day 3). The patch was removed between days 2 and 6 (median day 5) corresponding to an overlap with the full opioid in all cases (median 3 days, range 1 to 5 days). Initiation was completed within 1 week (median 5 days; range 4 to 7 days) and all patients fully transitioned from their full opioid agonists to buprenorphine monotherapy. Three cases achieved a buprenorphine dose of 16 mg or greater at the end of initiation. Four cases (57.1%) experienced withdrawal of any severity during the initiation process. No cases experienced moderate to severe withdrawal.

### Fentanyl patch bridging

One inpatient case used a fentanyl patch (25 mcg/hr for 5 days) to bridge to sublingual buprenorphine (see Additional file 2: Appendix Table S4). The buprenorphine doses began shortly after the fentanyl patch was removed, when the COWS score was zero. The initial buprenorphine dose was 1 mg with repeat doses of 1 mg and 2 mg to a total dose of 8 mg that day. No symptoms of withdrawal were reported during initiation but eight days post initiation the patient tested positive for opioids, buprenorphine, and methamphetamine and subsequently died from an overdose.

### Other

One case combined micro-dosing with buprenorphine patch bridging while 2 cases combined micro-dosing with Sustained Release Oral Morphine (SROM) bridging (see Additional file 2: Appendix Table S5). All cases occurred in the outpatient setting.

In the case evaluating combination micro-dosing and buprenorphine patch bridging, a 5mcg/hr buprenorphine patch was applied on day 1 and continued for days 2 and 3. On day 4, the patient transitioned to buprenorphine microdosing with an initial dose of 0.5 mg. The initiation process took 12 days and mild withdrawal was reported during initiation.

In the 2 cases evaluating combination micro-dosing and SROM bridging, initial doses of buprenorphine were 0.5 mg and 0.2 mg. Only one case was able to completely transition to buprenorphine monotherapy after 24 days and there were several protocol deviations that did lead to mild to moderate withdrawal. The second case did not complete the transition to buprenorphine and continued concurrent use of diacetylmorphine for > 250 days. Mild to moderate withdrawal symptoms occurred four times during initiation.

## Discussion

### Summary of findings

This systematic review aimed to evaluate the efficacy and safety of buprenorphine initiation strategies that omit the traditional approach of waiting for opioid withdrawal symptoms. Evidence is limited to case reports, no controlled studies were identified. The most common strategies reported in cases included microdosing and bridging with transdermal therapies. Approximately half of cases still reported withdrawal symptoms during initiation, albeit mild in severity. Given the lack of controlled studies, evidence is insufficient to determine the efficacy or safety of alternative initiation strategies presently.

Current case reports suggest it may be reasonable to expect some patients will successfully transition to sublingual buprenorphine with little to no symptoms of withdrawal while using an alternative approach, something that isn’t possible with traditional initiation of buprenorphine. On the other hand, initiation using alternative strategies may take longer to complete. Traditional initiation appears to be a quicker process (2–4 days) [23–25] whereas the median time to complete alternative initiation in the included cases was 1 week. Prior research suggests initiation that is too gradual is associated with high rates of drop-out among patients with OUD [24] and it is unclear if this would apply to these alternative strategies of initiating buprenorphine.

Despite a lack of current evidence, there are also practical challenges to implementation of alternative initiation strategies for buprenorphine. Insurance coverage for off-label use may be a barrier to successful implementation of certain strategies [26]. Traditional initiation typically occurs in the outpatient setting, whereas many of the included cases describe inpatient initiation which has direct implications to resources and costs of care. In general, many of the alternative initiation methods require manipulation of prescription products to achieve small enough doses to be consistent with published protocols. For example, manipulation of buprenorphine/naloxone to execute a microdosing protocol requires using scissors, razors, or folding and ripping buprenorphine/naloxone films to achieve the desired dose [27]. In the outpatient setting, patients are left to manipulate the dosage forms themselves. In the inpatient setting, some institutions may choose not to operationalize these practices based on the available evidence or may have policies and procedures that prohibit certain strategies [28, 29].

While eliminating prerequisite withdrawal is well intentioned, how it impacts the overall success of OUD treatment is left to be determined. For patients with OUD, these strategies may be a last line effort to initiate a mortality-reducing medication. As a result, there is a critical need for future research to inform the efficacy and safety of differing initiation strategies using buprenorphine. Anecdotal evidence suggests varying institutions may be already developing internal guidelines and protocols of novel dosing strategies, which further supports the need for validation [14, 30]. Ideally, studies are needed to compare alternative strategies to traditional initiation and include not only short-term outcomes of initiation, but also long-term indicators of success, such as retention, and mortality. Ongoing studies may begin to inform this topic soon (NCT04228250, NCT04234191).

For future directions, investigators should consider prospective randomized trials comparing novel dosing strategies to traditional buprenorphine initiation using validated tools to measure withdrawal outcomes. No comparative studies exist evaluating withdrawal severity between varying buprenorphine initiation strategies. While cases suggest alternative strategies may result in less withdrawal during the initiation process, this observation needs evaluation in a controlled, prospective trial. Additionally, investigators should take into consideration long term outcomes such as buprenorphine retention, which is approximately 45–50% at 24 weeks [31, 32].

Based on the data available, it may be reasonable to consider an alternative buprenorphine initiation strategy if patients cannot tolerate traditional prerequisite withdrawal and thus would otherwise preclude them from being a candidate for buprenorphine. While some patients did experience withdrawal with alternative strategies, most experienced mild symptoms and some experienced no withdrawal; this would be unattainable with traditional buprenorphine initiation. Buprenorphine microdosing and buprenorphine patch bridging were the most common strategies and were executed in patients with a wide variety of historical opioid use. Clinicians should be cognizant that the impact of omitting prerequisite withdrawal on long term outcomes is unknown.

### Limitations

We aimed to include all types of studies and despite this, only case reports and case series exist, which have very low internal and external validity. However, case reports can provide valuable insight into novel treatment strategies for a known condition that may be further explored [21]. We assessed internal validity of included cases and cannot rule out selection bias towards favorable outcomes because case selection was unclear. We were limited by reporting within the cases; at times we were unable to confirm the presence or absence of an outcome simply because there wasn’t enough information reported. We were left to rely on qualitative statements describing withdrawal outcomes in some cases, and it is unknown if these statements were founded on validated tools or were the opinion of the patient or provider. Finally, in order to focus on initiation strategies that omit traditional prerequisite withdrawal, we did not consider other possible strategies such as those that may use an opioid free window that is shorter than current recommendations, which may be an additional approach to balancing the benefits and risks of buprenorphine initiation.

## Conclusion

Alternative buprenorphine strategies that omit prerequisite withdrawal have emerged, however evidence is of low quality and limited to cases. Withdrawal symptoms were commonly reported, although mostly mild in severity. Without controlled studies, the efficacy and safety of these strategies in the setting of opioid use disorder treatment is unknown, leaving an unmet need for future research.

## Supplementary Information

**Additional file 1: Figure S1.** Database screening flowsheet.

**Additional file 2: Table S1.** Patient characteristics.** Table S2.** Summary of induction characteristics.** Appendix Table S1.** Quality assessment summary. **Appendix Table S2.** Induction phase characteristics of micro-dosing strategies. **Appendix Table S3.** Induction phase characteristics of buprenorphine patch bridging strategies. **Appendix Table S4.** Induction phase characteristics of fentanyl patch bridging strategies. **Appendix Table S5.** Induction phase characteristics of other strategies.

**Additional file 3. **Search strategy for systematic review.

## Data Availability

Datasets used and/or analyzed during the systematic review are available from the corresponding author on reasonable request.

## Abbreviations

COWS: Clinical Opiate Withdrawal Scale
SROM: Sustained Release Oral Morphine

## References

[1] Sordo L, Barrio G, Bravo MJ (2017). Mortality risk during and after opioid substitution treatment: systematic review and meta-analysis of cohort studies. BMJ.

[2] Auriacombe M, Fatséas M, Dubernet J, Daulouède JP, Tignol J (2004). French field experience with buprenorphine. Am J Addict.

[3] Walsh SL, Preston KL, Stitzer ML, Cone EJ, Bigelow GE (1994). Clinical pharmacology of buprenorphine: ceiling effects at high doses. Clin Pharmacol Ther.

[4] Kornfeld H, Reetz H (2015). Transdermal buprenorphine, opioid rotation to sublingual buprenorphine, and the avoidance of precipitated withdrawal: a review of the literature and demonstration in three chronic pain patients treated with butrans. Am J Ther.

[5] Virk MS, Arttamangkul S, Birdsong WT, Williams JT (2009). Buprenorphine is a weak partial agonist that inhibits opioid receptor desensitization. J Neurosci.

[6] Rosen K, Gutierrez A, Haller D, Potter JS (2014). Sublingual buprenorphine for chronic pain: a survey of clinician prescribing practices. Clin J Pain.

[7] Raheemullah A, Lembke A (2019). Initiating opioid agonist treatment for opioid use disorder in the inpatient setting: a teachable moment. JAMA Intern Med.

[8] Mattick RP, Ali R, White JM, O’brien S, Wolk S, Danz C (2003). Buprenorphine versus methadone maintenance therapy: a randomized double-blind trial with 405 opioid-dependent patients. Addiction.

[9] Kinasz KR, Herbst ED, Kalapatapu RK (2020). Case report: buprenorphine induction using transdermal buprenorphine in a veteran with opioid use disorder and psychosis, managing precipitated withdrawal. Mil Med.

[10] Mackie SE, Mchugh RK, Mcdermott K, Griffin ML, Winkelman JW, Weiss RD (2017). Prevalence of restless legs syndrome during detoxification from alcohol and opioids. J Subst Abuse Treat.

[11] Saal D, Lee F (2020). Rapid induction therapy for opioid-use disorder using buprenorphine transdermal patch: a case series. Perm J..

[12] Whitley SD, Sohler NL, Kunins HV (2010). Factors associated with complicated buprenorphine inductions. J Subst Abuse Treat.

[13] Surmaitis RM, Khalid MM, Vearrier D, Greenberg MI (2018). Takotsubo cardiomyopathy associated with buprenorphine precipitated withdrawal. Clin Toxicol (Phila).

[14] Randhawa PA, Brar R, Nolan S (2020). Buprenorphine-naloxone, “microdosing”: an alternative induction approach for the treatment of opioid use disorder in the wake of North America’s increasingly potent illicit drug market. CMAJ.

[15] The American Society of Addiction Medicine (ASAM): National Practice Guideline for the Treatment of Opioid Use Disorder. 2020 Focused Update.tment of Addiction Involving Opioid Use. https://www.asam.org/docs/default-source/quality-science/npg-jam-supplement.pdf?sfvrsn=a00a52c2_2. Published December 18, 2019. Accessed 28 Sept 2020.

[16] Hämmig R, Kemter A, Strasser J (2016). Use of microdoses for induction of buprenorphine treatment with overlapping full opioid agonist use: the Bernese method. Subst Abuse Rehabil.

[17] Moher D, Liberati A, Tetzlaff J, Altman DG (2009). Preferred reporting items for systematic reviews and meta-analyses: the PRISMA statement. BMJ.

[18] Wesson DR, Ling W (2003). The Clinical Opiate Withdrawal Scale (COWS). J Psychoact Drugs.

[19] The National Alliance of Advocates for Buprenorphine Treatment. Clinical Opiate Witdhrawal Scale (COWS). https://www.naabt.org/documents/cows_induction_flow_sheet.pdf. Accessed 28 Sept 2020.

[20] The American Society of Addiction Medicine (ASAM): National Practice Guideline for the Treatment of Opioid Use Disorder. 2020 Focused Update. Treatment of Addiction Involving Opioid Use. https://www.asam.org/docs/default-source/quality-science/npg-jam-supplement.pdf?sfvrsn=a00a52c2_2. Published December 18, 2019. Accessed 28 Sept 2020.

[21] Murad MH, Sultan S, Haffar S, Bazerbachi F (2018). Methodological quality and synthesis of case series and case reports. BMJ Evid Based Med.

[22] Fareed A, Vayalapalli S, Casarella J, Drexler K (2012). Effect of buprenorphine dose on treatment outcome. J Addict Dis.

[23] Dosing Guide for Optimal Management of Opioid Dependence. http://www.naabt.org/documents/Suboxone_Dosing_guide.pdf. Accessed 28 Sept 2020.

[24] Buprenorphine/naloxone sublingual film. Package insert. North Chesterfield, Virginia; 2019.

[25] American Society of Addiction Medicine (ASAM). Clinical Induction Protocol. https://www.asam.org/docs/default-source/education-docs/clinic-induction-protocol-example_it-matttrs_8-28-2017.pdf?sfvrsn=a30640c2_2. Published August 28, 2017. Accessed 28 Sept 2020.

[26] Criteria Based Consultation Prescribing Program Criteria for Drug Coverage Buprenorphine Transdermal Patch (Butrans®). Kaiser Permanente. https://healthy.kaiserpermanente.org/static/health/pdfs/formulary/nw/Butrans.pdf. Published September 2017. Accessed 28 Sept 2020.

[27] Reindel KL, Deangelis MJ, Ferrara AS (2019). An exploratory study of suboxone (buprenorphine/naloxone) film splitting: cutting methods, content uniformity, and stability. Int J Pharm Compd.

[28] Callan J, Pytell J, Ross J, Rastegar DA (2020). Transition from methadone to buprenorphine using a short-acting agonist bridge in the inpatient setting: a case study. J Addict Med.

[29] State Operations Manual: Regulations and Interpretive Guidelines for Hospitals. https://www.cms.gov/Regulations-and-Guidance/Guidance/Manuals/Downloads/som107ap_a_hospitals.pdf. February 21, 2020. Accessed 28 Sept 2020.

[30] Buprenorphine/Naloxone Microdosing: the Bernese method a brief summary for primary care clinicians. September 2019. https://metaphi.ca/assets/documents/provider%20tools/PCP_Microdosing_TVFHT.pdf. Accessed 14 Apr 2021.

[31] Teruya C, Schwartz RP, Mitchell SG (2014). Patient perspectives on buprenorphine/naloxone: a qualitative study of retention during the starting treatment with agonist replacement therapies (Start) study. J Psychoactive Drugs.

[32] Soyka M, Zingg C, Koller G, Kuefner H (2008). Retention rate and substance use in methadone and buprenorphine maintenance therapy and predictors of outcome: results from a randomized study. Int J Neuropsychopharmacol.

