# Supplementary material for: Initiating buprenorphine to treat opioid use disorder without prerequisite withdrawal: a systematic review
Source: Addict Sci Clin Pract. 2021 Jun 8;16:36. doi: 10.1186/s13722-021-00244-8 (PMC8186092; doi:10.1186/s13722-021-00244-8)
Supplement: Supplementary file 1 — Additional file 1: Figure S1. Database screening flowsheet. [file 13722_2021_244_MOESM1_ESM.pptx]

## Slide 1
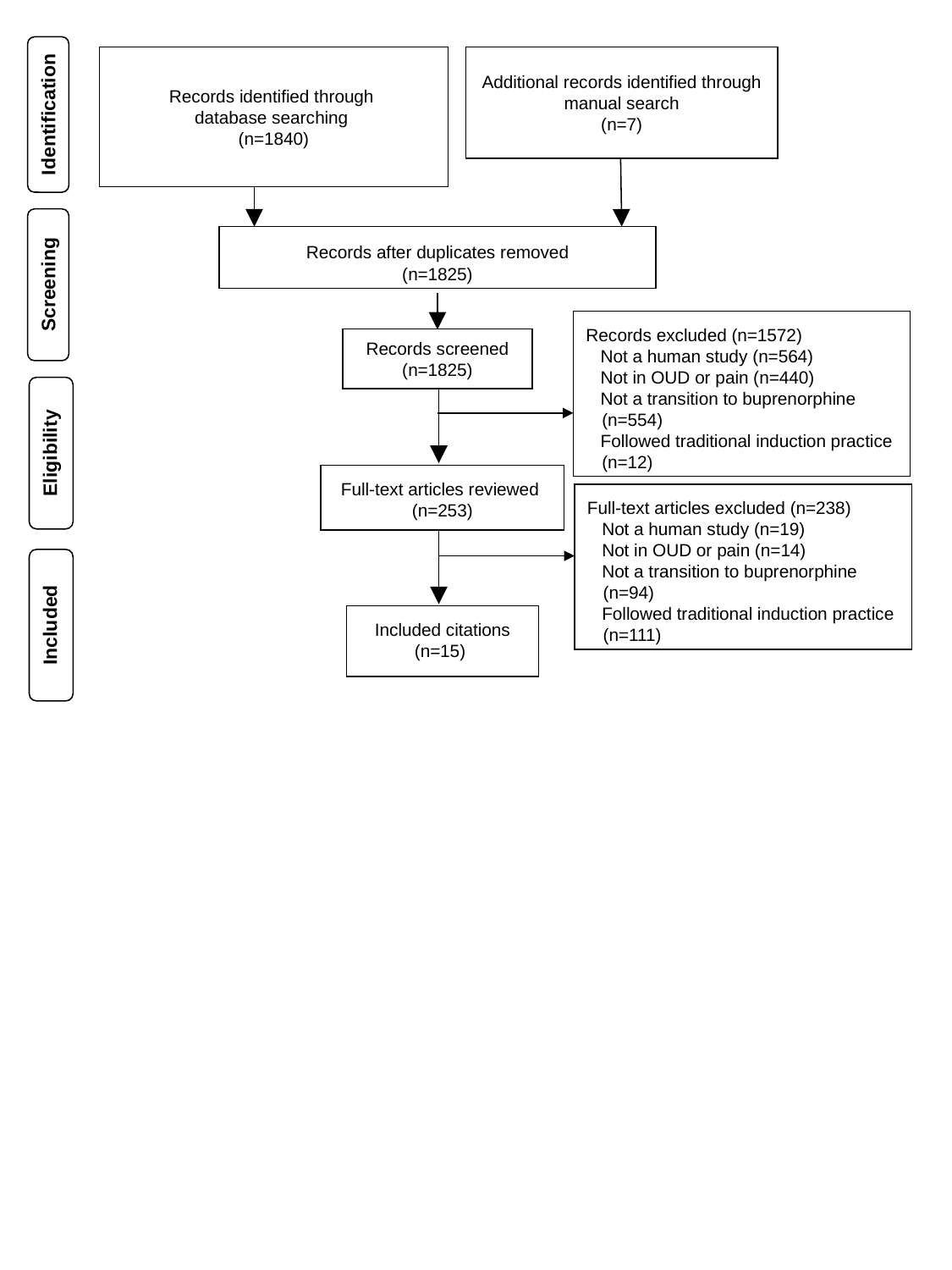

Records identified through
database searching
(n=1840)
Additional records identified through manual search
(n=7)
Identification
Records after duplicates removed
(n=1825)
Screening
Records excluded (n=1572)
 Not a human study (n=564)
 Not in OUD or pain (n=440)
 Not a transition to buprenorphine (n=554)
 Followed traditional induction practice (n=12)
Records screened
(n=1825)
Eligibility
Full-text articles reviewed
(n=253)
Full-text articles excluded (n=238)
 Not a human study (n=19)
 Not in OUD or pain (n=14)
 Not a transition to buprenorphine (n=94)
 Followed traditional induction practice (n=111)
Included
Included citations (n=15)
