# Supplementary material for: Initiating buprenorphine to treat opioid use disorder without prerequisite withdrawal: a systematic review
Source: Addict Sci Clin Pract. 2021 Jun 8;16:36. doi: 10.1186/s13722-021-00244-8 (PMC8186092; doi:10.1186/s13722-021-00244-8)
Supplement: Supplementary file 2 — Additional file 2: Table S1. Patient characteristics. Table S2. Summary of induction characteristics. Appendix Table S1. Quality assessment summary. Appendix Table S2. Induction phase characteristics of micro-dosing strategies. Appendix Table S3. Induction phase characteristics of buprenorphine patch bridging strategies. Appendix Table S4. Induction phase characteristics of fentanyl patch bridging strategies. Appendix Table S5. Induction phase characteristics of other strategies. [file 13722_2021_244_MOESM2_ESM.docx]

**Table S1. Patient characteristics**

| **Feature** | **N (%)** |
| --- | --- |
| Patient cases | 24 |
| Country |  |
| USA | 11 (45.8) |
| Canada | 10 (41.7) |
| Switzerland | 3 (12.5) |
| Male | 12^1^ (54.6) |
| Age (median, range) | 42.5 (19-72) |
| Indication for buprenorphine |  |
| OUD | 10 (41.7) |
| Pain | 4 (16.7) |
| Both | 10 (41.7) |
| Induction setting |  |
| Inpatient | 11 (45.8) |
| Outpatient | 13 (54.2) |
| Induction strategy |  |
| Micro-dosing |  |
| Bernese | 10 (41.7) |
| Rapid | 3 (12.5) |
| Bridge |  |
| Buprenorphine Patch | 7 (29.2) |
| Fentanyl Patch | 1 (4.2) |
| Other |  |
| Micro-dosing + bridge with  buprenorphine patch | 1 (4.2) |
| Micro-dosing + bridge with  SROM | 2 (8.3) |

Abbreviations: OUD=opioid use disorder; SROM=sustained-release

oral morphine; USA=United State of America

^1^Two cases did not report gender

**Table S2. Summary of induction characteristics**

| **Induction Strategy** | **No. of patients*** | **History of heroin use**  **n/N (%)** | **Completed transition to buprenorphine**  **n/N (%)** | **Time to complete induction,**  **median (range)** | **Patient deviation from induction,**  **n/N (%)** | **Any withdrawal during induction,**  **n/N (%)** | **Moderate or severe withdrawal during induction,**  **n/N (%)** |
| --- | --- | --- | --- | --- | --- | --- | --- |
| **Micro-dosing** | 13 | 9/13 (69.2) | 11/13 (84.6) | 8d  (3 to 120) | 2/2 (100) | 7/13 (53.8) | 1/12 (8.3) |
| **Buprenorphine patch bridging** | 7 | 2/7 (28.6) | 7/7 (100) | 5d  (4 to 7) | 2/2 (100) | 4/7 (57.1) | 0/6 (0) |
| **Fentanyl patch bridging** | 1 | 1/1 (100) | 1/1 (100) | 1d  (NA) | NR | 0/1 (0) | 0/1 (0) |
| **Other** | 3 | 3/3 (100) | 2/3 (66.6) | 24d  (12 to >129) | 1/1 (100) | 3/3 (100) | 1/2 (50) |

*The total number of cases is reported although not all cases provided enough information to determine if the listed outcome occurred or not, therefore were not included in the denominator for that outcome.

**Appendix Table S1. Quality Assessment Summary**

| **Author, year** | **Clear selection methods** | **Adequately ascertained exposure** | **Outcome adequately ascertained** | **Alternative causes of the observation ruled out** | **Follow-up long enough** | **Sufficient detail to replicate** |
| --- | --- | --- | --- | --- | --- | --- |
| Caulfield, 2020 | N | N | N | N | Y | Y |
| De Aquino, 2020 | N | N | N | N | Y | Y |
| Lee, 2020 | N | Y | Y | N | Y | Y |
| Rozylo, 2020 | N | N | N | N | Y | Y |
| Saal, 2020 | N | N | N | N | Y | Y |
| Jafari, 2019 | N | N | N | N | Y | Y |
| Klaire, 2019 | N | Y | Y | N | Y | Y |
| Martin, 2019 | N | Y | Y | N | Y | Y |
| Raheemullah, 2019 | N | Y | Y | N | Y | Y |
| Sandhu, 2019 | N | Y | Y | N | Y | Y |
| Terasaki, 2019 | N | Y | Y | N | Y | Y |
| Vogel, 2019 | N | N | N | N | Y | Y |
| Azar, 2018 | N | Y | Y | N | Y | Y |
| Hämmig, 2016 | N | N | N | N | Y | Y |
| Kornfeld, 2015 | N | N | N | N | Y | N |

**Appendix Table S2. Induction phase characteristics of micro-dosing strategies**

| **Author, Case #** | **History of opioid use** | **Opioid regimen prior to induction** | **Initial buprenorphine dose, (mg)** | **Overlap time** | **Time to complete induction** | **Buprenorphine dose at the end of induction (SL mg/d)** | **Ancillary medications**† |
| --- | --- | --- | --- | --- | --- | --- | --- |
| Hämmig, R, Case 1 | Heroin | Heroin | 0.2 | 5d | 9d | 12 | NR |
| Hämmig, R, Case 2 | Diacetylmorphine, methadone, heroin | Methadone moderate dose + Diacetylmorphine | 0.2 | 28d | 29d | 24 | NR |
| Jafari, S Case 1 | Methadone, illicit opioids | Methadone high dose | 0.5 | 4m | 4m | 32 | Clonidine |
| Klaire, S Case 1 | Heroin | Heroin + short acting opioids | 0.25 | 4d | 5d | 16 | NR |
| Klaire, S Case 2 | Heroin | Short acting opioids | 0.5 | 2d | 3d | 12 | NR |
| Lee, D Case 1 | Short acting opioids | Methadone low dose + short acting opioids | 0.25 | 4d | 5d | 16 | Clonidine, APAP, pregabalin, methadone, |
| Martin, L Case 1 | Methadone and buprenorphine | Short acting opioids | 0.5 | 12d | 14d | 10 | NR |
| Martin L, Case 2 | Short acting opioids | Short acting opioids | 0.5 | NA* | NA* | NA* | NR |
| Rozylo, J Case 1 | Heroin, methadone | Heroin | 0.25 | NA* | NA* | NA* | NR |
| Sandhu, R Case 1 | Short acting opioids, heroin | Short acting opioids | 0.25 | 6d | 7d | 12 | APAP |
| Terasaki, D Case 1 | Heroin, buprenorphine | Methadone low dose | 0.5 | 7d | 8d | 12 | NR |
| Terasaki, D Case 2 | Heroin, methadone | Methadone moderate dose + short acting opioids | 0.5 | 7d | 11d | 24 | NR |
| Terasaki, D Case 3 | Heroin | Methadone low dose + short acting opioids | 0.5 | 7d | 8d | 12 | NR |

Abbreviations: APAP=acetaminophen; d=day; m=month; mg=milligram; NA=not applicable; NR=not reported; SL=sublingual

*Did not complete induction and remained on concurrent full agonist therapy as adjunct to buprenorphine

†Medications that alleviate physical symptoms of opioid withdrawal or pain

**Appendix Table S3. Induction phase characteristics of buprenorphine patch bridging strategies**

| **Author, Case #** | **History of opioid use** | **Opioid regimen prior to induction** | **Strength of buprenorphine patch (ug/hr)** | **Time of physical overlap** | **Time to complete induction** | **Buprenorphine dose at the end of induction (SL mg/d)** | **Ancillary medications**† |
| --- | --- | --- | --- | --- | --- | --- | --- |
| Kornfeld, H Case 1 | Long acting + short acting opioids | Short acting opioids | 20 | 1d | NR | 32 | Benzodiazepines, NSAIDs, pregabalin |
| Kornfeld, H Case 3 | Methadone + short acting opioids + long acting opioids | Short acting opioids | 20 | NR | NR | 32 | Benzodiazepines, APAP |
| Raheemullah, A Case 1 | Heroin | 220mg morphine equivalents* | 20 | 3d | 4d | 10 | NR |
| Saal, D Case 2 | Short acting opioids; buprenorphine use | Short acting opioids | 10 | 5d | 6d | 16 | NR |
| Saal, D Case 3 | Heroin; methadone | Kratom | 10 | 4d | 5d | 8 | NR |
| Saal, D Case 4 | Long acting opioids + short acting opioids | Long acting opioids + short acting opioids | 10 | 3d | 7d | 4 | NR |
| Saal, D Case 5 | Short acting opioids; buprenorphine use | Short acting opioids | 10 | 2d | 5d | 8 | NR |

Abbreviations: APAP=acetaminophen; d=day; hr=hour; mg=milligram; NR=not reported; NSAID=non-steroidal anti-inflammatory; SL=sublingual; ug=microgram

*specific regimen was not reported

†Medications that alleviate physical symptoms of opioid withdrawal or pain

**Appendix Table S4. Induction phase characteristics of fentanyl patch bridging strategies**

| **Author, Case #** | **History of opioid use** | **Opioid regimen prior to transition to fentanyl patch** | **Strength of fentanyl patch (ug/hr)** | **Time of physical overlap** | **Time to complete induction** | **Buprenorphine dose at the end of induction ( SL mg/d)** | **Ancillary medications**† |
| --- | --- | --- | --- | --- | --- | --- | --- |
| Azar, P Case 1 | Heroin; methadone | Methadone low dose + heroin | 25 | 0 | 1d | 8 | APAP |

Abbreviations: APAP=acetaminophen; d=day; hr=hour; mg=milligram; SL=sublingual; ug=microgram

†Medications that alleviate physical symptoms of opioid withdrawal or pain

**Appendix Table S5. Induction phase characteristics of other strategies**

| **Author, Case #** | **History of opioid use** | **Opioid regimen prior to induction** | **Bridge strategy** | **Sublingual buprenorphine dosing strategy** | **Time to complete induction** | **Buprenorphine dose at the end of induction (SL mg/d)** | **Ancillary medications**† |
| --- | --- | --- | --- | --- | --- | --- | --- |
| Caulfield, MDG Case 1 | Heroin; methadone and buprenorphine use | Short acting opioids + SROM | SROM | Micro-dosing | 24d | 16 | NR |
| De Aquino, J Case 1 | Methadone; heroin use; buprenorphine use | Methadone moderate dose | Buprenorphine patch | Micro-dosing | 12d | 16 | Clonidine, Loperamide, NSAIDs |
| Vogel, M Case 1 | Diacetylmorphine + methadone; heroin use | Diacetylmorphine + SROM | SROM | Micro-dosing | > 129d | NA* | NR |

Abbreviations: d=day; NA=not applicable; NR=not reported; NSAID=non-steroidal anti=inflammatory; SL=sublingual; SROM=sustained released oral morphine

*Did not complete induction and remained on concurrent full agonist therapy as adjunct to buprenorphine

†Medications that alleviate physical symptoms of opioid withdrawal or pain
