# Supplementary material for: Initiating buprenorphine to treat opioid use disorder without prerequisite withdrawal: a systematic review
Source: Addict Sci Clin Pract. 2021 Jun 8;16:36. doi: 10.1186/s13722-021-00244-8 (PMC8186092; doi:10.1186/s13722-021-00244-8)
Supplement: Supplementary file 3 — Additional file 3. Search strategy for systematic review. [file 13722_2021_244_MOESM3_ESM.docx]

Search strategy used in OVID to search MEDLINE

1. Chronic Pain/
2. Opioid-Related Disorders/
3. Opiate Substitution Treatment/
4. opioid use disorder.mp.
5. opioid dependence.mp.
6. opioid abuse.mp.
7. Heroin Dependence/
8. opiate use disorder.mp.
9. opiate abuse.mp.
10. opiate disorder.mp.
11. Receptors, Opioid, mu/
12. 1 or 2 or 3 or 4 or 5 or 6 or 7 or 8 or 9 or 10 or 11
13. Buprenorphine, Naloxone Drug Combination/ or Buprenorphine/ or buprenorphine.mp.
14. initiat*.mp.
15. microdos*.mp.
16. induc*.mp.
17. bridg*.mp.
18. replac*.mp.
19. substitut*.mp.
20. 14 or 15 or 16 or 17 or 18 or 19
21. 12 and 13 and 20
22. Limit 21 to animals
23. 21 not 22

22 Sub 12 and 20 and 21 2260 Advanced More

23 Sub limit 22 to animals [Limit not valid in CCTR; records were retained] 519 Advanced More

24 Sub 22 not 23
